# Changes in Influenza Activity and Circulating Subtypes During the COVID-19 Outbreak in China

**DOI:** 10.3389/fmed.2022.829799

**Published:** 2022-03-22

**Authors:** Luyan Zheng, Jinjin Qi, Jie Wu, Min Zheng

**Affiliations:** State Key Laboratory for Diagnosis and Treatment of Infectious Diseases, National Clinical Research Center for Infectious Diseases, Collaborative Innovation Center for Diagnosis and Treatment of Infectious Diseases, The First Affiliated Hospital, College of Medicine, Zhejiang University, Hangzhou, China

**Keywords:** COVID-19, influenza activity, influenza season, subtypes, non-pharmaceutical interventions

## Abstract

**Background:**

Non-pharmaceutical interventions (NPIs) to mitigate COVID-19 can impact the circulation of influenza viruses. There is a need to describe the activity of influenza and its subtypes during the COVID-19 pandemic to aid in the development of influenza prevention and control measures in the next influenza season.

**Method:**

Data from pathogenic surveillance performed by the Chinese National Influenza Center from January 2016 to August 2021 were extracted and stratified by type and subtype for northern China and southern China. The distribution of influenza activity and circulating subtypes were described during the COVID-19 pandemic, and data from 2016 to 2019 were used for comparisons.

**Results:**

Influenza activity declined rapidly and then rose slowly during the COVID-19 pandemic in China. The distribution of influenza subtypes changed from A-dominant to B/Victoria-dominant after the COVID-19 outbreak.

**Discussion:**

Whether the B/Yamagata lineage has disappeared from China deserves more attention in future virologic monitoring programs. The influenza vaccination campaign in the 2021–2022 season is an important means by which to reduce the proportion of susceptible people and limit the damage that potentially greater and earlier circulation of the virus could cause.

## Introduction

Since the worldwide outbreak of new coronavirus disease (COVID-19) in late 2019, non-pharmaceutical interventions (NPIs) have been proposed to prevent the spread of severe acute respiratory syndrome coronavirus 2 (SARS-CoV-2). Government-mandated NPIs mainly included city lockdown, mask-wearing, handwashing, quarantine, travel restrictions, and social distancing. Some of them (such as mask-wearing and travel restrictions) remained lifted in China almost 2 years after the outbreak. Previous studies indicated that surgical mask use could significantly reduce infection rates of SARS-CoV-2 (1), and the symptoms of infection were usually much milder among patients who used face masks (2). Quarantining and maintaining social distancing were also effective in decreasing COVID-19 cases (3).

Severe acute respiratory syndrome coronavirus 2 is a highly contagious respiratory virus that is mainly transmitted through droplets and aerosols by infected individuals to susceptible populations. The mode of transmission is consistent with that of seasonal influenza viruses. Changes in influenza virus circulation because of widespread implementation of measures to mitigate transmission of SARS-CoV-2 deserved extensive explorations (4). Indeed, one study conducted in March 2020, using data from the National Institute of Infectious Diseases Japan, showed that seasonal influenza activity was lower in the 2019–2020 influenza season than in previous seasons in Japan (5). Another study conducted in the United States, Australia, Chile, and South Africa has also demonstrated a decline in the activity of seasonal influenza during the COVID-19 pandemic (4).

It is worth noting that after 2 years of the emergence of COVID-19, the epidemic was largely controlled, but the circulation of influenza viruses did not increase to pre-pandemic levels. However, previous studies were mainly conducted in the early stage of COVID-19 epidemic. In addition, due to the city lockdown and quarantine, immunizations in all age groups, including influenza vaccination, have been interrupted, delayed, or completely suspended (6–8). Although the health consequences for delayed vaccination remained unclear, an influenza pandemic will inevitably be concurrent with or follow the COVID-19 outbreak if COVID-19 mitigation practices become less stringent and no action is taken. Health professionals should be sensitive to the irregular changes in the prevalence of seasonal influenza, and influenza vaccination remains an effective means by which to reduce the circulation of influenza viruses. Our goal was to describe the activity of influenza and its subtypes after the COVID-19 pandemic in China, and we used data from 2016 to 2019 for comparisons.

## Materials and Methods

### Data Source

The Chinese government established a national influenza center in 1957, which collected data available from the 31 provinces in mainland China. The Chinese National Influenza Center is affiliated with the National Institution for Viral Disease Control and Prevention, the Chinese Center for Disease Control and Prevention (CDC), and covers 554 influenza surveillance sentinel hospitals and 410 influenza surveillance network laboratories (9). In 2010, the Chinese National Influenza Center was appointed as the Collaborating Center for Reference and Research on Influenza by the World Health Organization (WHO); it was the fifth in the world and the only one covering developing areas. China’s current influenza surveillance is web-based and includes six domains: pathogenic surveillance, influenza-like illness (ILI) case reports, influenza outbreak surveillance, outbreaks of animal-derived influenza virus infection in humans, animal avian influenza outbreaks, and influenza surveillance in other countries and regions. The respiratory specimens of each ILI, who had a temperature ≥38.0°C and either cough or sore throat, were collected in sentinel hospitals and tested in influenza surveillance network laboratories (9). The types and subtypes of influenza viruses were confirmed by hemagglutination assays (HA) and hemagglutination inhibition (HI) assays, which were performed as usual during the COVID-19 pandemic.

### Procedures

Data were extracted from the China CDC Weekly report on influenza surveillance, which is publicly available from the data resource center of the Chinese National Influenza Center (10). Data extraction was conducted by one author (QJ) and double-checked by another author (ZL). Although there are four types of influenza viruses, A, B, C, and D, only influenza A and B viruses cause seasonal epidemics of disease (known as the flu season) and are included in the influenza surveillance weekly report (10). Influenza A viruses are divided into two subtypes (H1N1 and H3N2), while influenza B viruses are further classified into two lineages (Yamagata and Victoria) based on antigenic analyses (10). Moreover, pathogenic surveillance data are also divided into northern China (15 provinces) and southern China (16 provinces). Thus, data for pathogenic surveillance from January 2016 to August 2021 were obtained and stratified by type and subtype for northern China and for southern China.

### Statistical Analysis

We defined influenza activity as the percentage of respiratory specimens submitted for influenza testing that yielded positive results, which is a method that has been widely adopted in previous studies (11, 12). We grouped the weekly reports into seasons (week 34 of the year through week 33 of its following year; the season was truncated after week 33 in 2021 because this was the latest available data for 2021) and then compared the weekly influenza activity in each season. We also described the seasonal distribution with curves and bar graphs for weekly influenza activity, and the data from 2016 to 2019 were used for comparisons. In addition, we described the distribution of weekly influenza activity by subtype for northern China and for southern China, respectively. All statistical analyses and graph generation were performed with Python software, version 3.9 (Python Software Foundation).

## Results

**FIGURE 1:**
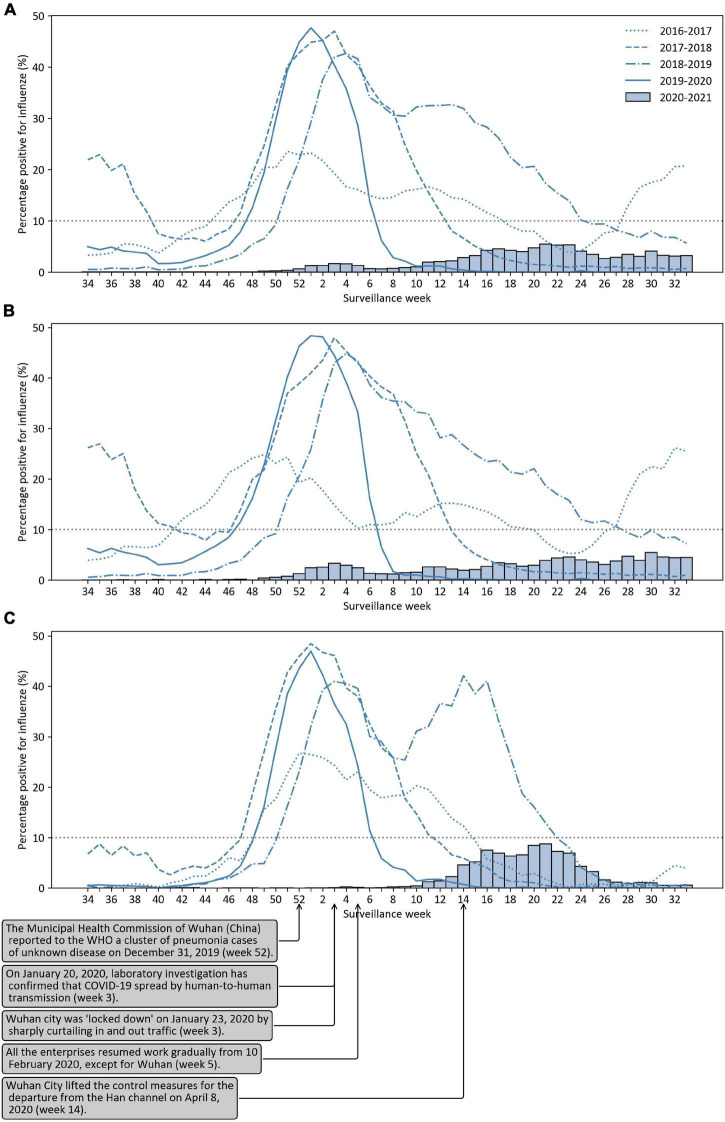
The percentage of positive tests for influenza viruses in the 2016–2017 through 2020–2021 seasons. **(A)** For mainland China; **(B)** For southern China; **(C)** For northern China.

Figure 1A shows the influenza activity observed in mainland China in five epidemiological seasons for the 2016–2017 through 2020–2021 seasons. In the 2019–2020 season, influenza activity peaked at 48%, with similar maximum values of influenza activity from the previous two seasons (2017–2018 season: 47%; 2018–2019 season: 42%). However, we observed that the peak influenza activity in the 2019–2020 season occurred approximately 2–3 weeks earlier than that observed in the previous two seasons, and influenza activity then rapidly declined. Influenza activity had dropped to less than 10% after February 21, 2020 (surveillance week 7–10: 1∼6%), which was significantly lower than that in the same period in any previous influenza season (surveillance week 7–10: 27∼33% in 2016–2017 season, 20∼33% in 2017–2018 season, 30∼32% in 2018–2019 season). Second, in the 2020–2021 season, the influenza activity peak (surveillance week 23: 5%) was much later and lower than in any previous influenza season since 2016. In fact, in the 2020–2021 influenza season, the rates of influenza-positive detection per surveillance week were all below 10%. During the 2020–2021 season, a total of 409,742 respiratory specimens were tested, which was higher than the number tested during the four previous seasons, but only 7,316 (2%) were positive for influenza, which was significantly lower than the positivity rate for each of the four previous seasons (2016–2017 season: 13%; 2017–2018 season: 18%; 2018–2019 season: 18%; 2019–2020 season: 12%) (Table 1).

**TABLE 1:** Influenza activity in mainland China, 2016–2017 through 2020–2021 seasons.

Influenza season	No. of respiratory specimens tested	No. of tested positive for influenza	Detection rates of tested positive	Influenza A			Influenza B		
				H3N2	H1N1	Aunsubtyped	Victoria	Yamagata	Bunsubtyped
2016–2017	276,191	36,375	13.17%	24,178	7,652	380	3,176	662	363
2017–2018	296,165	53,480	18.06%	10,809	19,433	170	2,682	19,754	634
2018–2019	319,501	57,525	18.00%	11,551	29,810	84	15,556	242	282
2019–2020	330,390	38,267	11.58%	24,503	2,817	28	10,717	58	144
2020–2021	409,742	7,316	1.79%	10	22	9	7,156	29	90

Figures 1B,C shows the influenza activity observed in southern China and northern China for the 2016–2017 through 2020–2021 seasons, respectively. In the 2019–2020 season, influenza activity peaked at 48% in the first week of 2020 and dropped down to 7% in the 7th surveillance week of 2020 in southern China. In northern China, influenza activity peaked at 47% in the first week of 2020 and dropped down to 6% in the 7th surveillance week of 2020 in the 2019–2020 season. In the 2020–2021 season, influenza activity peaked at 5 and 9% in southern China and northern China, respectively. Influenza activity in the southern provinces started to rise at the end of 2020 (week 49). However, influenza activity in the northern provinces started to rise in February 2021 (week 8) and declined from June 2021 (week 22) onward.

Table 1 shows the distribution of influenza A subtypes and influenza B lineages of respiratory specimens. In China, influenza A was more prevalent than B in the 2019–2020 season, which was a trend also observed in three previous influenza seasons. In the 2016–2017 through 2019–2020 seasons, public health laboratories tested 1,222,247 specimens, and 185,647 (15.2%) were positive for influenza; among these specimens, 131,415 (70.8%) were positive for influenza A virus, and 54,270 (29.2%) were positive for influenza B viruses. However, in the 2020–2021 season, influenza B activity was significantly higher than influenza A activity. During this period, the number of respiratory specimens submitted for influenza testing that yielded positive results was 7,316; 41 (0.6%) were positive for influenza A virus, and 7,275 (99.4%) were positive for influenza B viruses. Of the 7,185 influenza B viruses with B lineage results, 7,156 (99.6%) were B/Victoria, and 29 (0.4%) were B/Yamagata.

Tables 2, 3 shows the distribution of influenza subtypes of influenza-positive specimens in southern China and northern China from 2016 to 2021, respectively. We observed that the distribution of influenza subtypes was dominated by influenza A in both southern China and northern China from 2016–2017 influenza season to 2019–2020 influenza season. In the 2020–2021 season, however, a low distribution of influenza A and B/Yamagata viruses during the COVID–19 pandemic was observed in both southern China and northern China. During this period, the number of respiratory specimens submitted for influenza testing that yielded positive results was 5,024 in southern China; 30 (0.6%) were positive for influenza A virus, and 4,994 (99.4%) were positive for influenza B viruses. Of the 4,994 influenza B viruses with B lineage results, 4,916 (98.4%) were B/Victoria, and 18 (0.4%) were B/Yamagata (Table 2). In northern China, the number of respiratory specimens submitted for influenza testing that yielded positive results was 2,292 in the 2020–2021 season; 11 (0.5%) were positive for influenza A virus, and 2,281 (99.5%) were positive for influenza B viruses. Of the 2,281 influenza B viruses with B lineage results, 2,240 (98.2%) were B/Victoria, and 11 (0.5%) were B/Yamagata (Table 3).

**TABLE 2:** Influenza activity in Southern China, 2016–2017 through 2020–2021 seasons.

Influenza season	No. of respiratory specimens tested	No. of tested positive for influenza	Detection rates of tested positive	Influenza A			Influenza B		
				H3N2	H1N1	Aunsubtyped	Victoria	Yamagata	Bunsubtyped
2016–2017	172,105	23,701	13.77%	15,986	3,555	280	3,031	546	304
2017–2018	173,977	29,662	17.05%	5,874	11,085	98	2,260	9,945	384
2018–2019	188,370	32,649	17.33%	6,682	16,290	60	10,225	159	233
2019–2020	198,756	21,127	10.63%	11,058	1,222	17	8,676	23	131
2020–2021	240,683	5,024	2.09%	4	18	8	4,916	18	60

**TABLE 3:** Influenza activity in Northern China, 2016–2017 through 2020–2021 seasons.

Influenza season	No. of respiratory specimens tested	No. of tested positive for influenza	Detection rates of tested positive	Influenza A			Influenza B		
				H3N2	H1N1	Aunsubtyped	Victoria	Yamagata	Bunsubtyped
2016–2017	104,086	12,710	12.21%	8,192	4,096	102	145	116	59
2017–2018	122,474	23,818	19.45%	4,935	8,348	72	422	9,810	232
2018–2019	131,131	24,876	18.97%	4,869	14,520	24	5,331	83	49
2019–2020	131,634	17,140	13.02%	13,445	1,594	11	2,041	35	13
2020–2021	168,859	2,292	1.36%	6	4	1	2,240	11	30

## Discussion

In China, influenza virus circulation declined sharply within 8 weeks of the COVID-19 emergency declaration and the widespread implementation of community mitigation measures. After the COVID-19 outbreak was brought under control, influenza activity increased but was substantially lower than in previous seasons, before the COVID-19 epidemic. Moreover, before the COVID-19 outbreak, the distribution of influenza subtypes was dominated by influenza A, whereas after the COVID-19 outbreak, the distribution of influenza subtypes was dominated by influenza B.

The COVID-19 outbreak led to an earlier end of the influenza season and reduced influenza activity; then, after the COVID-19 outbreak was under control, influenza activity increased slowly bur remained at a low level. This phenomenon has been described previously in the United States (4, 13). In the initial stage of COVID-19 outbreak, declines in influenza virus activity were attributed to decreased testing, because people with respiratory symptoms were often referred for SARS-CoV-2 testing. However, it has been confirmed that NPIs implemented to mitigate COVID-19 can partially mitigate seasonal pandemic influenza (14). Like SARS-CoV-2, influenza viruses are spread primarily by droplet transmission, the widespread NPIs, including school closures, social distancing, and mask wearing, may substantially reduce the possibility of influenza exposure risk. Although causality cannot be inferred from our study, the consistent trends over time and places are compelling and biologically plausible. These findings suggest that NPIs might be useful adjuncts to influenza vaccination during influenza seasons.

It is notable that there were also some differences between and changes in influenza activity in China and the United States or other countries during the COVID-19 epidemic. First, influenza activity declined much earlier in China (peaked at week 1 and decreased sharply) than in the United States (peaked at week 6 and decreased sharply) in the 2019–2020 flu season (4). This might be because China was the first country that took strict measures to respond to COVID-19. Second, in the 2020–2021 season, influenza activity in China (1.8%) was higher than that in the United States (0.2%) (13). Notably, influenza activity in China was usually higher than that in the United States in the pre-COVID-19 influenza seasons. This could be attributable to the lower influenza vaccination rate in China and different testing practices for influenza virus between countries. In the 2018–2019 season, the overall influenza vaccination rate was 2.4 and 57.9% in China (15) and the United States (16), respectively, which might have resulted in a lower protection rate in the Chinese population together with an accumulation of susceptible people (17). Overall, vaccination remains one of the best interventions to prevent influenza (18), but certain community mitigation measures might be useful adjuncts to influenza vaccination, particularly for populations with contraindications to vaccination.

Before the COVID-19 outbreak, influenza A viruses dominated in both southern and northern China, whereas after the COVID-19 outbreak, influenza B viruses dominated, 99.7% of which were B/Victoria viruses (B/Yamagata: 0.03%) in the 2020–2021 season. Decreased influenza A cases and pseudo-extinction of B/Yamagata viruses during the COVID-19 pandemic have been reported elsewhere (19). The explanation for this phenomenon is unclear. However, it is presumed that this may be related to differences in susceptible populations. Influenza A (both H1N1 and H3N2) and influenza B/Yamagata are known to disproportionately affect adults and older adults, who are also more susceptible to COVID-19, whereas children are more susceptible to B/Victoria viruses (20, 21). Adults are also more affected by current NPIs, especially by the restrictions on social gathering and by the disruption in travel. Therefore, the transmission of influenza A (both H1N1 and H3N2) and influenza B/Yamagata was significantly disrupted when strict NPIs were implemented. This shift in influenza type and subtype dominance may have important implications for influenza prevention. Given the lack of sustained natural exposure to certain influenza virus type, the reduction in the circulation of influenza viruses over the past year may affect the severity of the upcoming influenza season.

The findings in this report are subject to at least three limitations. First, the analysis included only virological surveillance data, which might be affected by positive specimen collection rates and case selection biases. Second, this was an ecologic analysis that cannot demonstrate causality, and we cannot rule out the effects of unmeasured or residual confounding. Third, there are many factors that can interrupt the transmission of influenza viruses and reduce influenza activity during the COVID-19 pandemic, including restrictions of personal movement (e.g., keeping a safe social distance, restricting international travel, and issuing stay-at-home orders), and use of individual protection (e.g., wearing masks, reinforced hygiene, and reducing the gathering of crowds), but these were not assessed.

## Conclusion

Here, we provide evidence that current NPIs have been tremendously effective in limiting the spread of influenza. A shift in influenza virus type and subtype dominance should also be highlighted. Whether the B/Yamagata lineage disappears from China deserves more attention in future virologic monitoring programs. However, it is necessary to consider that non-exposure to certain pathogens may have unanticipated consequences in the future. One of the most important measures in the face of declining immunity is the development of an “influenza booster strategy” in the 2021–2022 vaccination campaign as a means to artificially reduce the proportion of susceptible people and thus to limit the damage that potentially greater and earlier circulation of the influenza virus could cause.

## Data Availability Statement

The original data used in the study are available at: https://ivdc.chinacdc.cn/cnic/zyzx/, further inquiries can be directed to the corresponding authors.

## Author Contributions

MZ conceived and designed the study assisted by JW. JQ and LZ acquired the data. LZ performed analyses assisted by JW. LZ drafted the manuscript and all authors critically revised it. All authors assume full responsibility for the accuracy and completeness of the ideas presented.

## Conflict of Interest

The authors declare that the research was conducted in the absence of any commercial or financial relationships that could be construed as a potential conflict of interest.

## Publisher’s Note

All claims expressed in this article are solely those of the authors and do not necessarily represent those of their affiliated organizations, or those of the publisher, the editors and the reviewers. Any product that may be evaluated in this article, or claim that may be made by its manufacturer, is not guaranteed or endorsed by the publisher.
